# Identification of novel vascular markers through gene expression profiling of tumor-derived endothelium

**DOI:** 10.1186/1471-2164-9-201

**Published:** 2008-04-30

**Authors:** Carmen Ghilardi, Giovanna Chiorino, Romina Dossi, Zsuzsanna Nagy, Raffaella Giavazzi, MariaRosa Bani

**Affiliations:** 1 Laboratory of Biology and Treatment of Metastases, Mario Negri Institute for Pharmacological Research, Milano, Italy; 2 Laboratory of Cancer Genomics, Fondo "Edo Tempia", Biella, Italy; 3 Neuroscience Division, Medical School, University of Birmingham, Birmingham, B15 2TT, UK

## Abstract

**Background:**

Targeting tumor angiogenesis and vasculature is a promising strategy for the inhibition of tumor growth and dissemination. Evidence suggests that tumor vasculature expresses unique markers that distinguish it from normal vasculature. Our efforts focused on the molecular characterization of endothelial cells (EC) in the search for selective markers of tumor vasculature that might be helpful for the development of effective therapeutic approaches.

**Results:**

We investigated by microarray analysis the gene expression profiles of EC purified and cultured from tumor (ovarian carcinoma [HOC-EC]) and normal (human adrenal gland [HA-EC]) tissue specimens. We found distinct transcriptional features characterizing the EC of different origin, and identified 158 transcripts highly expressed by HOC-EC. We analyzed four of these genes, ADAM23, FAP, GPNMB and PRSS3, which were not previously known to be expressed by endothelium. *In vitro *experiments confirmed the higher expression of the selected genes in tumor-derived endothelium with no expression in tumor cells. *In vivo *investigation by *in situ *hybridization established that ADAM23, GPNMB and PRSS3 expression is localized on blood vessels of human cancer specimens.

**Conclusion:**

These findings elucidate some of the molecular features of the tumor endothelium. Comparative transcriptomic analysis allowed us to determine molecular differences of tumor and normal tissue-derived endothelium and to identify novel markers that might be exploited to selectively target tumor vasculature.

## Background

Interference with tumor vessel formation and blood supply has become a well-recognized approach of cancer therapy [1], as epitomized by the recent FDA approval of antiangiogenic drugs such as the humanized anti Vascular Endothelial Growth Factor (VEGF) antibody Avastin^® ^[2]. Vascularization is required for tumor growth and metastasis, and constitutes an important step in the control of cancer progression [3]. Experimental evidence correlates tumor vascularization with high malignancy and poor prognosis, and shows that elevated levels of angiogenic factors, such as VEGF and basic Fibroblast Growth Factor (FGF-2), are associated with tumor progression [4]. Moreover, expansive growth of metastasis appears to be linked to the ability to stimulate endothelial cell growth [5].

Tumor-induced vessels are ultrastructurally abnormal and differ from normal vasculature. They lack functional pericytes and are unusually permeable [6]. These abnormalities reflect the pathological nature of their induction, and underpin the novel therapeutic strategies directed against the vascular elements of the tumor stroma to selectively affect tumor vasculature and inhibit tumor growth [7].

In recent years an increasing body of evidence suggests that tumor vasculature expresses unique markers that distinguish it from normal vasculature. Numerous efforts have been aimed at the molecular characterization of tumor associated endothelial cells (EC) in the search for differences between tumor and normal tissue EC. A variety of techniques have been applied to study EC *in vivo*, isolated from *ex vivo *specimens or cultured *in vitro*. The *in vivo *phage display technology was used to identify molecules selectively expressed on tumor endothelium [8,9], while other approaches exploited the *in vivo *proteomic analysis to detect antigens accessible from the vasculature [10,11]. These techniques have allowed, mostly through studies conducted in experimental tumor models, the identification of vascular-specific motifs expressed by different tumor types and during different stages of carcinogenesis.

Gene expression of EC isolated from *ex vivo *human cancer and normal specimens, analyzed by means of Serial Analysis of Gene Expression (SAGE) or microarray, revealed differences at the transcriptional level in tumor and normal tissues [12-15]. *In vitro *models have been widely used to identify the specific functions of the endothelium and to understand the molecular modifications that might occur during angiogenesis. In these studies EC were exposed to different microenvironmental conditions, such as angiogenic growth factors and matrix proteins, shear stress, hypoxia, or tumor conditioned media [16-20]. All of these approaches led to the identification of mechanisms and molecules that are potentially involved in the formation of blood vessels. Despite these findings, attempts to discover tumor EC markers have always been hampered by technical difficulties in isolating functionally intact and phenotypically stable EC from tumor tissues. In fact, all the *in vitro *models have used cultures of immortalized EC and/or primary EC from human umbilical vein (HUVEC).

We developed a method to isolate and culture EC from tumor specimens (TdEC), by which we were able to show that TdEC *in vitro *maintain several of the features described for tumor vasculature and that they differ from EC isolated from normal tissue (i.e., from human adrenal gland HA-EC) [21]. These findings suggested that TdEC and HA-EC might represent useful tools to study tumor vessel properties and, ultimately, to identify tumor vasculature markers.

Here we describe the isolation of EC from human specimens and the characterization of the transcriptional profiles of EC derived from ovarian carcinoma (HOC-EC) and HA-EC by means of microarray technology. Through the comparison of HOC-EC and HA-EC gene expression we were able to demonstrate that tumor-associated EC differ from those in normal tissues, thus proving that molecular differences are maintained *in vitro*. We successfully identified molecules that are expressed selectively by the tumor vasculature *in vivo*, thus providing distinctive features to be exploited to selectively target tumor vasculature.

## Results

### Characterization of Endothelial Cells (EC) isolated from human specimens

Endothelial cells were reproducibly isolated from human tissue specimens: HOC-EC from ovarian carcinoma, HKC-EC from kidney carcinoma, HA-EC from adrenal gland and HSk-EC from skin.

Before use, their endothelial origin was established. Endothelial cell cultures typically created a homogeneous monolayer of elongated cells in close contact and maintained an uniform morphology after few passages in culture; they formed capillary-like cord structures when plated on matrigel (data not shown and [21]). Their endothelial origin was established by positive immunostaining for platelet-endothelial cell adhesion molecule-1 (CD31/PECAM-1), von Willebrand Factor (vWF) and vascular E-cadherin (VE-cadherin) (Table 1). In addition, no staining was observed when the cells were analyzed for the expression of alpha-Smooth Muscle Actin (alpha-SMA), a typical non-endothelial marker (Table 1). Flow cytometry studies assessed the ability to uptake low density lipoproteins (LDL), a characteristic of endothelial cells, and confirmed the positivity for CD31/PECAM-1 (data not shown).

**Table 1:** Typical endothelial signatures of the cells isolated from human cancer and normal tissue specimens

	**CD31/PECAM-1**	vWF*	**VE-cadherin**	**alpha-SMA**
**HOC-EC **(n = 9)	73–98	75–99	97–100	0
**HKC-EC **(n = 3)	85–93	27–67	nt	0
**HA-EC **(n = 9)	94–99	30–90	99–100	0
**HSk-EC **(n = 2)	95–97	80–99	nt	0
**HUVEC **(n = 6)	80–99	90–99	99–100	0

Percentages of positive cells analyzed by immunohistochemistry evaluating a total of 500 cells/population. In parentheses are the number of independent human EC populations evaluated (HOC-EC = ovarian carcinoma EC; HKC-EC = kidney carcinoma EC; HA-EC = adrenal gland-EC; HSk-EC = skin-EC, HUVEC = umbilical vein EC).

Shown are the range of values obtained for the different endothelial cell types.

* Population showing positivity values below 65% were not utilized. nt=not determined

The purity of the isolated EC was comparable to that of EC from umbilical vein (HUVEC), which were used as the reference population for EC (Table 1).

Microarray results, performed on HA-EC and HOC-EC, confirmed the endothelial origin of the isolated cells (Table 2). The typical endothelial genes expressed at high levels include COL8A1, COL18A1, CYR61, endoglin, E-selectin, HIF1A, ICAM-1, JAM3 junctional adhesion molecule, neuropilin-2, PAR-1 thrombin receptor, P-selectin, TEK endothelial tyrosine kinase, VEGFR-1, and VEGFR-2. Among these, the presence of transcripts for CD31/PECAM-1, VE-cadherin and vWF, whose protein expression was shown by immunohistochemical methods (Table 1), is worth emphasizing.

**Table 2:** Typical endothelial genes highly expressed by HOC-EC and HA-EC

**Unigene Cluster**	**Symbol**	**Gene Name**
Hs.83347	AAMP	Angio-associated, migratory cell protein
Hs.426312	AMOTL2	Angiomotin like 2
Hs.521731	ANGPTL2	Angiopoietin-like 2
Hs.448589	ANKRD1	Ankyrin repeat domain 1 (cardiac muscle)
Hs.185055	BENE	BENE protein
Hs.495731	BMX	BMX non-receptor tyrosine kinase
Hs.76206	CDH5	Cadherin 5, type 2, VE-cadherin (vascular epithelium)
Hs.482562	F2R	Coagulation factor II (thrombin) receptor, PAR-1
Hs.134830	COL8A1	Collagen, type VIII, alpha 1
Hs.517356	COL18A1	Collagen, type XVIII, alpha 1
Hs.410037	CTGF	Connective tissue growth factor
Hs.8867	CYR61	Cysteine-rich, angiogenicinducer, 61
Hs.174050	EDF1	Endothelial differentiation-related factor 1
Hs.76753	ENG	Endoglin (Osler-Rendu-Weber syndrome 1)
Hs.129944	ESM1	Endothelial cell-specific molecule 1
Hs.154210	EDG1	Endothelial differentiation, sphingolipid G-protein-coupled receptor, 1
Hs.122575	EDG4	Endothelial differentiation, lysophosphatidicacid G-protein-coupled receptor, 4
Hs.468410	EPAS1	Endothelial PAS domain protein 1
Hs.507621	FLT-1	Fms-related tyrosine kinase 1
Hs.367725	GATA2	GATA binding protein 2
Hs.201712	GLG1	Golgi apparatus protein 1
Hs.509554	HIF1A	Hypoxia-inducible factor 1, alpha subunit
Hs.515126	ICAM-1	Intercellular adhesion molecule 1 (CD54)
Hs.150718	JAM3	Junctional adhesion molecule 3
Hs.479756	KDR	Kinase insert domain receptor
Hs.268107	MMRN1	Multimerin 1
Hs.471200	NRP2	Neuropilin 2
Hs.511603	NOS3	Nitric oxide synthase3
Hs.514412	PECAM1	Platelet/endothelial cell adhesion molecule (CD31 antigen)
Hs.405156	PPAP2B	Phosphatidic acid phosphatase type 2B
Hs.252820	PGF	Placental growth factor, vascular endothelial growth factor-related protein
Hs.82353	PROCR	Protein C receptor, endothelial (EPCR)
Hs.89546	SELE	Selectin E (endothelial adhesion molecule 1)
Hs.73800	SELP	Selectin P (granule membrane protein 140kDa, antigen CD62)
Hs.514913	SERPINB2	Serine (or cysteine) proteinaseinhibitor, clade B (ovalbumin), member 2
Hs.414795	SERPINE1	Serine (or cysteine) proteinaseinhibitor, clade E, member 1
Hs.89640	TEK	TEK tyrosine kinase, endothelial
Hs.50382	TJP2	Tight junction protein 2 (zona occludens 2)
Hs.76090	TNFAIP1	Tumor necrosis factor, alpha-induced protein 1(endothelial)
Hs.525607	TNFAIP2	Tumor necrosis factor, alpha-induced protein 2
Hs.440848	VWF	Von Willebrand factor

Three HOC-EC and two HA-EC populations were investigated by Affymetrix GeneChip^® ^Human Genome U95A Arrays. This list of highly expressed endothelial genes was extracted from the microarray results by keywords search within the complete probeset list annotated using SOURCE batch search (see Methods for details).

### Genome wide transcriptional differences: tumor vs. normal tissue derived EC

Comparison of the transcriptional profiles of HOC-EC and HA-EC (experimental design and analyses detailed in Methods) showed that EC from tumor and normal tissue have distinct and characteristic expression patterns that are maintained *in vitro*. According to our selection criteria, 158 gene transcripts, corresponding to 179 probe sets, were more expressed by HOC-EC than by HA-EC. The genes belong to the different functional classes that are listed in Table 3.

**Table 3:** Transcriptional differences: tumor vs normal tissue derived EC

**Unigene Cluster**	**Symbol**	**Gene Name**	
**Structural**			A
Hs.370287	ADAM23	A disintegrin and metalloproteinase domain 23	
Hs.118127	ACTC	Actin, alpha, cardiac muscle	
Hs.533336	BAMBI	BMP and activin membrane-bound inhibitor homolog	
Hs.489142	COL1A2	Collagen, type I, alpha 2	
Hs.443625	COL3A1	Collagen, type III, alpha 1	
Hs.210283	COL5A1	Collagen, type V, alpha 1	
Hs.211933	COL13A1	Collagen, type XIII, alpha 1	
Hs.409034	COL15A1	Collagen, type XV, alpha 1	
Hs.80552	DPT	Dermatopontin	
Hs.412597	DSG2	Desmoglein 2	
Hs.159291	DRP2	Dystrophin related protein 2	
Hs.371903	GYPE	Glycophorin E	
Hs.190495	GPNMB	Glycoprotein (transmembrane) nmb	
Hs.328232	GPC1	Glypican 1	
Hs.435557	KIF5C	Kinesin family member 5C	
Hs.436367	LAMA3	Laminin, alpha 3	
Hs.519972	HLA-F	Major histocompatibility complex, class I, F	
Hs.490874	MTX1	Metaxin 1	
Hs.464469	MYOM1	Myomesin 1 (skelemin)	
Hs.504687	MYL9	Myosin, light polypeptide 9, regulatory	
Hs.464829	CDH2	N-cadherin	
Hs.116471	CDH11	OB-cadherin	
Hs.487925	PDE4DIP	Phosphodiesterase 4D interacting protein (myomegalin)	
Hs.157818	KCNAB1	Potassium voltage-gated channel, shaker-related subfamily, beta member 1	
Hs.407643	PCDH9	Protocadherin 9	
Hs.106511	PCDH17	Hypothetical protein LOC144997	
Hs.7972	RIPX	Rap2 interacting protein x	
Hs.73800	SELP	Selectin P	
Hs.468675	T1A-2	T1A-2 (lung type-I cell membrane-associated glycoprotein)	
Hs.143250	TNC	Tenascin C	
Hs.371147	THBS2	Thrombospondin 2	
Hs.443681	CSPG2	Versican	
Hs.529901	XIST	X (inactive)-specific transcript	

**Enzymatic**			B
Hs.459538	ALDH1A3	Aldehyde dehydrogenase 1 family, member A3	
Hs.421202	ABCA2	ATP-binding cassette, sub-family A (ABC1), member 2	
Hs.489033	ABCB1	ATP-binding cassette, sub-family B (MDR/TAP), member 1	
Hs.646	CPA3	Carboxypeptidase A3	
Hs.75360	CPE	Carboxypeptidase E	
Hs.252549	CTSZ	Cathepsin Z	
Hs.154654	CYP1B1	Cytochrome P450, family 1, subfamily B, polypeptide 1	
Hs.516493	FAP	Fibroblast activation protein, alpha	
Hs.30332	GFPT2	Glutamine-fructose-6-phosphate transaminase 2	
Hs.301961	GSTM1	Glutathione S-transferase M1	
Hs.470126	KYNU	Kynureninase (L-kynurenine hydrolase)	
Hs.65436	LOXL1	Lysyl oxidase-like 1	
Hs.307734	MME	Membrane metallo-endopeptidase	
Hs.478289	NLGN1	Neuroligin 1	
Hs.30954	PMVK	Phosphomevalonate kinase	
Hs.77274	PLAU	Plasminogen activator, urokinase	
Hs.446429	PTGDS	Prostaglandin D2 synthase 21 kDa	
Hs.501280	PRSS11	Protease, serine, 11 (IGF binding)	
Hs.303090	PPP1R3C	Protein phosphatase 1, regulatory (inhibitor) subunit 3C	
Hs.128013	PRSS3	Protease, serine, 3	
Hs.270279	TYRP1	Tyrosinase-related protein 1	
Hs.13225	B4GALT4	UDP-Gal:betaGlcNAc beta 1,4- galactosyltransferase, polypeptide 4	
Hs.69009	B3GNT3	UDP-GlcNAc:betaGal beta-1,3-N-acetylglucosaminyltransferase 3	

**Signaling**			C
Hs.143102	AB012943	Amine oxidase, copper containing 2	
Hs.167046	ADORA2B	Adenosine A2b receptor	
Hs.483909	ADMR	Adrenomedullin receptor	
Hs.132902	CAP2	CAP, adenylate cyclase-associated protein, 2 (yeast)	
Hs.502328	CD44	CD44 antigen	
Hs.78065	C7	Complement component 7	
Hs.473133	DOK5	Docking protein 5	
Hs.523173	ENTPD1	Ectonucleoside triphosphate diphosphohydrolase 1	
Hs.511899	EDN1	Endothelin 1	
Hs.1407	EDN2	Endothelin 2	
Hs.183713	EDNRA	Endothelin receptor type A	
Hs.481371	FAT	FAT tumor suppressor homolog 1 (Drosophila)	
Hs.352	FOLR3	Folate receptor 3 (gamma)	
Hs.432395	GPR39	G protein-coupled receptor 39	
Hs.79022	GEM	GTP binding protein overexpressed in skeletal muscle	
Hs.198612	GPR51	G protein-coupled receptor 51	
Hs.156855	GABRG3	Gamma-aminobutyric acid (GABA) A receptor, gamma 3	
Hs.167017	GABBR1	Gamma-aminobutyric acid (GABA) B receptor, 1	
Hs.98262	GRIK2	Glutamate receptor, ionotropic, kainate 2	
Hs.124161	HCN2	Hyperpolarization activated cyclic nucleotide-gated potassium channel 2	
Hs.370984	IGSF4C	Immunoglobulin superfamily, member 4C	
Hs.438102	IGFBP2	Insulin-like growth factor binding protein 2	
Hs.369982	IGFBP5	Insulin-like growth factor binding protein 5	
Hs.81134	IL1RN	Interleukin 1 receptor antagonist	
Hs.126256	IL1B	Interleukin 1, beta	
Hs.129751	IL17R	Interleukin 17 receptor	
Hs.514535	LGALS3BP	Lectin, galactoside-binding, soluble, 3 binding protein	
Hs.125474	LPXN	Leupaxin	
Hs.36566	LIMK1	LIM domain kinase 1	
Hs.304475	LCP2	Lymphocyte cytosolic protein 2 (SH2 domain containing leukocyte protein of 76 kDa)	
Hs.418367	NMU	Neuromedin U	
Hs.514556	NPTX1	Neuronal pentraxin I	
Hs.8546	NOTCH3	Notch homolog 3 (Drosophila)	
Hs.434255	PSD3	Pleckstrin and Sec7 domain containing 3	
Hs.506076	PTPRR	Protein tyrosine phosphatase, receptor type, R	
Hs.436456	ROR1	Receptor tyrosine kinase-like orphan receptor 1	
Hs.24950	RGS5	Regulator of G-protein signalling 5	
Hs.194691	RAI3	Retinoic acid induced 3	
Hs.95655	SECTM1	Secreted and transmembrane 1	
Hs.516726	SCG2	Secretogranin II (chromogranin C)	
Hs.156540	SGNE1	Secretory granule, neuroendocrine protein 1 (7B2 protein)	
Hs.75149	SH3GL2	SH3-domain GRB2-like 2	
Hs.444915	SLC1A1	Solute carrier family 1, member 1	
Hs.448520	SLC7A2	Solute carrier family 7, member 2	
Hs.351306	SLC16A4	Solute carrier family 16, member 4	
Hs.192686	SLC20A2	Solute carrier family 20, member 2	
Hs.12409	SST	Somatostatin	
Hs.62886	SPARCL1	SPARC-like 1 (mast9, hevin)	
Hs.233160	STC2	Stanniocalcin 2	
Hs.258326	LOC51760	Synaptotagmin 1	
Hs.349470	SNCG	Synuclein, gamma	
Hs.62192	TF	Tissue factor	
Hs.534363	UCN	Urocortin	
Hs.73793	VEGF	Vascular endothelial growth factor	
Hs.306251	ERBB3	V-erb-b2 erythroblastic leukemia viral oncogene homolog 3 (avian)	

**Transcriptional factors and regulators**			D
Hs.65734	ARNTL	Aryl hydrocarbon receptor nuclear translocator-like	
Hs.397705	CAMTA1	Calmodulin binding transcription activator 1	
Hs.157002	DZIP1	DAZ interacting protein 1	
Hs.22634	ETV1	ets variant gene 1	
Hs.155591	FOXF1	Forkhead box F1	
Hs.519385	FOXD1	Forkhead related activator 4 (FREAC-4)	
Hs.121443	HOP	Homeodomain-only protein	
Hs.180919	ID2	Inhibitor of DNA binding 2, dominant negative helix-loop-helix protein	
Hs.371013	JMJD2B	Jumonji domain containing 2B	
Hs.434284	LRRC17	Leucine rich repeat containing 17	
Hs.527007	MEOX2	Mesenchyme homeo box 2 (growth arrest-specific homeo box)	
Hs.89404	MSX2	Msh homeo box homolog 2 (Drosophila)	
Hs.1497	RARG	Retinoic acid receptor, gamma	
Hs.149261	RUNX1	Runt-related transcription factor 1 (acute myeloid leukemia 1; aml1 oncogene)	
Hs.465087	SMAD7	SMAD, mothers against DPP homolog 7 (Drosophila)	
Hs.360174	SNAI2	Snail homolog 2 (Drosophila)	
Hs.89583	TLX1	T-cell leukemia, homeobox 1	
Hs.149991	THOC2	THO complex 2	
Hs.125962	TFEC	Transcription factor EC	
Hs.1145	WT1	Wilms tumor 1	
Hs.490510	ZNF212	Zinc finger protein 212	
Hs.22653	ZNF365	Zinc finger protein 365	
Hs.147765	ZNF415	Zinc finger protein 415	

**DNA replication, Cell Cycle and Apoptosis**			E
Hs.75823	AF1Q	ALL1-fused gene from chromosome 1q	
Hs.150749	BCL2	B-cell CLL/lymphoma 2	
Hs.122908	CDT1	DNA replication factor	
Hs.9999	EMP3	Epithelial membrane protein 3	
Hs.52903	8FSTL3	Follistatin-like 3 (secreted glycoprotein)	
Hs.130853	HIST1H2BD	Histone 1, H2bd	
Hs.534369	HIST1H2BE	Histone 1, H2be	
Hs.152944	LOH11CR2A	Loss of heterozygosity, 11, chromosomal region 2, gene A	
Hs.432132	G0S2	Putative lymphocyte G0/G1 switch gene	
Hs.514913	SERPINB2	Serine (or cysteine) proteinase inhibitor, clade B (ovalbumin), member 2	

**Others and unknown**			F
Hs.441783	C14orf78	C14orf78	
Hs.516956	C20orf91	chromosome 20 open reading frame 91 Hs.17936	
	DKFZP434H132	DKFZP434H132 protein	
Hs.132994	DKFZP434C171	DKFZP434C171 protein	
Hs.41707	HSPB3	Heat shock 27 kDa protein 3	
Hs.432616	IMP-3	IGF-II mRNA-binding protein 3	
Hs.534372	INE1	Inactivation escape 1	
Hs.464563	KIAA0802	KIAA0802	
Hs.65750	KIAA1086	KIAA1086	
Hs.472010	PRNP	Prion protein (p27-30)	
	AC006276		
	U62317		
	W26626		
	W28546		

List of the 158 gene transcipts more expressed by HOC-EC than HA-EC.

Three HOC-EC and two HA-EC populations were investigated by Affymetrix GeneChip^® ^Human Genome U95A Arrays and analyzed searchingfor intrinsic differences, as described in Methods.

Microarray results reveal an alteration in cell-cell and cell-matrix interaction as shown by the enhanced expression of molecules such as desmoglein 2, N- and OB-cadherin, protocadherin-9 and -17 and P-selectin (Table 3A). In keeping with the notion that the extracellular matrix in tumor differs from that in normal tissue [22] glypican-1, laminin alpha 3, tenascin C, versican, as well as different types of collagens (collagen typeI-alpha2, typeIII-alpha1, typeV-alpha1, typeXIII-alpha1 and typeXV-alpha1) and thrombospondin-2, were expressed at higher levels by HOC-EC than by HA-EC (Table 3A). Moreover, alterations of cytoskeletal protein involved in cell motility, for instance the actin and myosin related molecules MYOM1, MYL9 and RIPX, were also observed (Table 3A).

The peptidase family is the most represented among the enzymatic classes of genes expressed by HOC-EC (Table 3B). It includes carboxypeptidase A3, carboxypeptidase E, cathepsin Z, membrane metallo-endopeptidase, serinprotease 11, and urokinase-plasminogen activator. This is consistent with the concept that peptidases are very important for the activation of pro-enzymes or protein precursors, as well as in the degradation of the extracellular matrix [23].

Most of the listed genes are involved in signaling and comprise molecules previously shown to be involved in angiogenesis and other vascular functions, such as adrenomedullin receptor, CD44, endothelin-1 and -2, endothelin receptor-A and tissue factor (Table 3C).

Consistent with the notion that the vascular and nervous systems share similar signals and principles [24], 3 members of the GABA receptors family and several molecules involved in synaptic signaling, as well as elements involved in brain differentiation and axon elongation, were expressed at higher levels by HOC-EC (Table 3C).

Four members of the Solute Carrier Family (SLC1A1, SLC7A2, SLC16A4, SLC20A2) were over-expressed in HOC-EC, suggesting an alteration in the extra-intracellular exchange of metabolites (Table 3C). Among the transporters, the enhanced expression of the ATP-binding cassette family members ABCA2 and ABCB1 (Table 3B) should be emphasized. ABCB1, best known as MDR/p-glycoprotein, is involved in drug removal from cells.

Higher expression also affected genes involved in transcriptional response, such as members of the zinc finger protein family (DZIP1, JMJD2B, SNAI2, WT1, ZNF212, ZNF365, ZNF415) and of the homeobox family (HOP, MEOX2, MSX2, TLX1) (Table 3D).

SMAD7 (Table 3D) and BAMBI (Table 3A), involved and acting as inhibitors of TGF-beta signaling, were also more expressed.

Among the class of cell cycle regulators, the high expression of the anti-apoptotic protein BCL2 is noteworthy (Table 3E).

### Selected transcripts are expressed by tumor derived endothelial cells

In order to identify novel markers of tumor endothelium our attention focused on transcripts that are highly expressed by tumor-derived endothelium (i.e., HOC-EC), but whose products and functions were not previously reported as being involved in tumor angiogenesis or associated to vascular functions. Among them we distinguished four genes on the basis of their structural or functional characteristics, such as the presence of transmembrane domains and/or enzymatic activities: disintegrin and metalloproteinase domain 23 (ADAM23), fibroblast activation protein alpha (FAP), transmembrane glycoprotein nmb (GPNMB), and serine protease 3 or mesotrypsin (PRSS3). The relative expression in HOC-EC compared to HA-EC was 2.6 for ADAM23, 4.1 for FAP, 4.6 for GPNMB and 2.5 for PRSS3, as calculated from microarray data. These results were validated by real-time PCR in EC isolated from six neoplastic tissues (five ovarian and one kidney carcinoma specimens) and from four non-neoplastic tissues (three adrenal glands and one skin specimen) different from those investigated by microarray. These tumor or normal tissue derived EC are described in Table 1.

**Figure 1:**
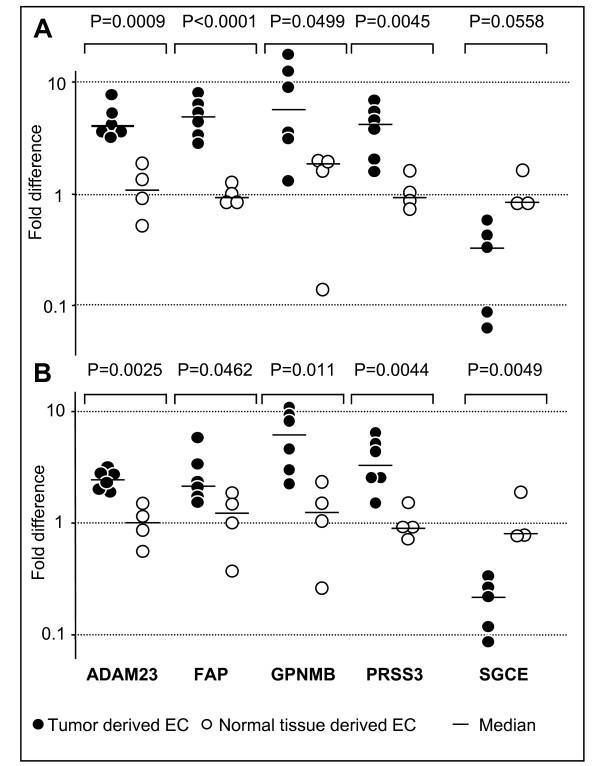
**Real-time PCR quantification of gene expression in endothelial cells isolated from human cancer and normal tissue specimens**. Endothelial cells were exposed (A) or not (B) to VEGF, EGF, FGF-2 and fibronectin (see Methods for details). Fold differences for each EC population and p values for each gene analyzed are shown. The expression of the target gene was normalized to 18s rRNA for each of the EC populations being evaluated: ΔCt = Ct_target _- Ct_18s_. Statistical analysis on ΔCt values was performed comparing tumor and normal tissue derived EC. Fold differences were calculated according to the comparative ΔΔCt method: Fold difference = 2 ^-(ΔCt each population-ΔCt ref) ^by arbitrarily considering the normal tissue derived ECs as reference (ΔCt_ref _being their average ΔCt value).

To mimic the different environments in which EC might be embedded *in vivo *– either subjected to persistent stimulation imposed by the tumor cells or not, EC were investigated both in the presence (Figure 1A) or the absence (Figure 1B) of the angiogenic factors VEGF, FGF- 2, of epidermal growth factor (EGF) and of fibronectin. Under both conditions the expression of ADAM23, FAP, GPNMB and PRSS3 genes was much higher in EC isolated from tumor specimens, as shown by the fold difference values (Figures 1A and 1B). As specified by the p values, statistical analysis demonstrated a significant disparity in the expression levels for each of the genes being investigated (Figures 1A and 1B).

These results confirm microarray findings and demonstrate that such a diversity is indeed an intrinsic characteristic associated to the tumor or normal tissue origin of the EC, regardless of the culture conditions. Sarcoglycan epsilon (SCGE), chosen as an internal control of a gene expressed to a much lesser extent by HOC-EC, confirmed the microarray results (Figures 1A and 1B).

### Expression in non endothelial cells

**Figure 2:**
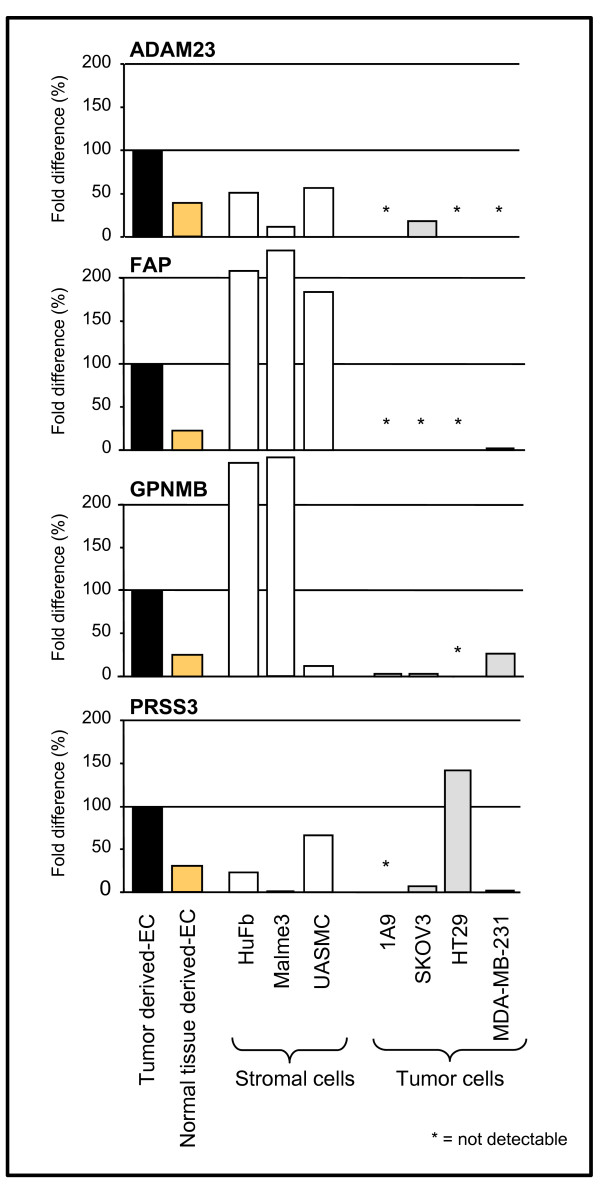
**Gene expression in different cell lines**. Levels of expression of ADAM23, FAP, GPNMB and PRSS3 were evaluated in fibroblasts (HuFb and Malme3) and smooth muscle cells (USMAC) as well as in tumor cell lines (1A9, SKOV3, HT29 and MDA-MB-231). Gene expression of each target gene was normalized to 18s rRNA for each cell type (ΔCt = Ct_target _- Ct_18s_). Average of ΔCt of the target gene from tumor derived ECs (Figure 1) was assumed as reference (ΔCt_ref_). Fold differences for each cell type were calculated according to the comparative ΔΔCt method (Fold difference = 2^-(ΔCt each celltype-ΔCt ref)^) and expressed as percentage relative to tumor-derived EC (100%).

**Figure 3:**
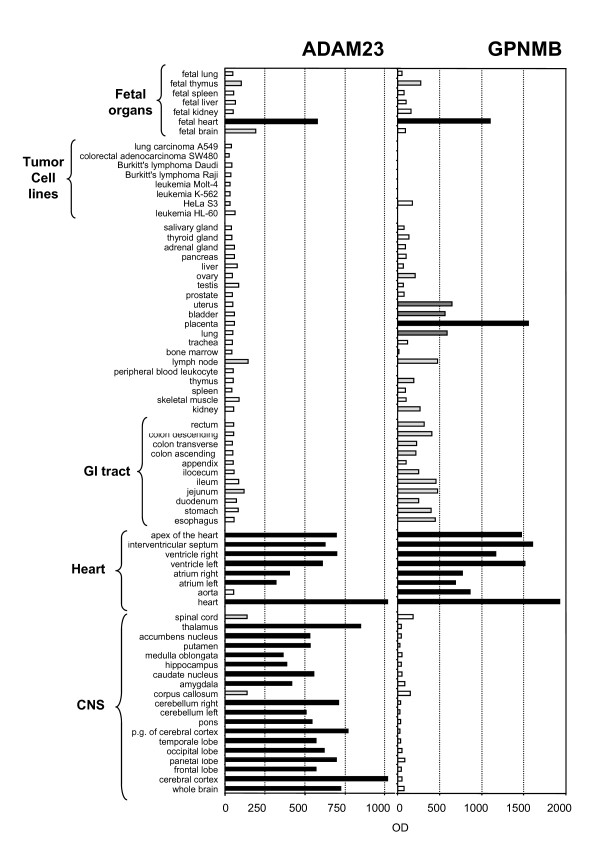
**Tissue expression of ADAM23 and GPNMB**. ADAM23 and GPNMB expression were evaluated by Multiple Tissue Expression array. The membrane, comprising polyA+ mRNA from different adult and fetal tissues, was hybridized with a ^32^P labeled probe specific for the two genes and hybridization visualized by autoradiography. Bars represent the optical density evaluated for each dot.

Having shown that ADAM23, FAP, GPNMB, and PRSS3 were expressed by a panel of EC, with the highest expression found in TdEC, we next investigated their expression in other cell types. The gene expression analysis revealed that they could be expressed to different extents also by other stromal cells, such as fibroblasts (HuFb and Malme3) and smooth muscle cells (UASMC), but in general not by tumor cells (Figure 2). Specifically, their expression in the carcinoma cell lines (1A9 and SKOV3 ovarian, MDA-MB-431 breast and HT29 colon) was not or was barely detectable (with the exception of PRSS3 by the HT29 colon carcinoma) (Figure 2). This lack of expression was supported by the Northern Blot results of a panel of tumor cell lines represented on the Multiple Tissue Expression array (Figure 3). Moreover, the Reference database for gene Expression Analysis (RefExA [25]) confirmed that the tumor cell lines did not show expression of the selected genes.

Conversely, the expression of FAP and GPNMB was higher in both the HuFb and MALME 3 fibroblasts than in EC, and FAP was also expressed at a higher level by the UASMC smooth muscle cells. ADAM23 and PRSS3 were instead expressed to a lesser extent by all the stromal cells analyzed (Figure 2).

### Expression in healthy tissues

Tissue expression of ADAM23 and GPNMB was assessed using a Multiple Tissue Expression (MTE) array: a panel of human mRNAs in dot-blot format from 58 normal adult tissues, seven fetal tissues, and eight cell lines. As shown in Figure 3, ADAM23 and GPNMB were expressed by a limited subset of tissues, while tumor cells did not express either one of them. In particular, ADAM23 expression was highest in cardiovascular tissues and fetal heart, as well as in central nervous system (CNS) samples, while it was much lower in fetal brain (Figure 3). The expression of GPNMB was highest in cardiovascular tissues and fetal heart, as well as in placenta, while it was detected to a much lesser extent in lung, uterus, and bladder tissue. (Figure 3). These Northern Blot results for ADAM23 and GPNMB expression were confirmed by the RefExA database [25].

Data from RefExA [25] and literature report the expression of PRSS3 by brain, colon and pancreas [26], while FAP expression is described only in the activated stromal fibroblast of tumors [27].

### Expression by tumor vasculature in vivo

In order to investigate the *in vivo *localization of ADAM23, GPNMB and PRSS3, human tumor tissues were analyzed by *in situ *hybridization.

**Figure 4:**
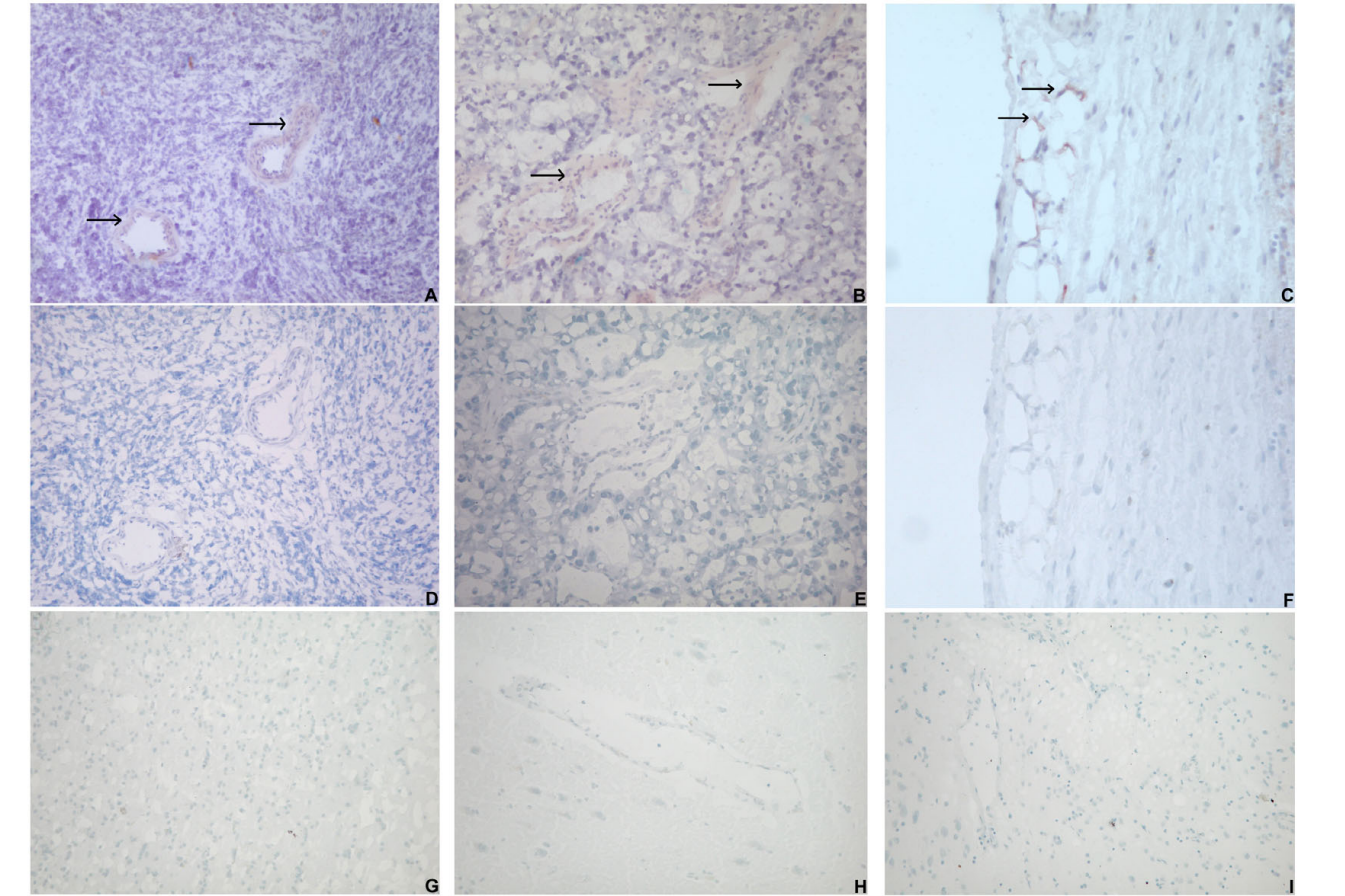
***In situ *hybridization of human tissues**. Shown are representative samples of medulloblastoma (A), brain metastasis of an adenocarcinoma (B) and Ewing sarcoma (C), hybridized with antisense probes for ADAM23, GPNMB and PRSS3, respectively. Positively stained blood vessels are indicated by arrows. Related hybridization with control sense probes for ADAM23, GPNMB and PRSS3 is shown in panel D-E-F. Normal brain samples, from different donors, hybridized respectively with antisense probes for ADAM23 (G) and GPNMB (H) are shown. Alzheimer's-diseased brain hybridized with antisense probe for PRSS3 is shown (I).

Endothelial expression of the selected genes was confirmed in the tumor samples analyzed. As shown in Figure 4, the hybridization signals for ADAM23, GPNMB, and PRSS3 localized around the blood vessels, and exclusively in the vascular walls. According to the *in vitro *results, tumor cells did not express the transcripts, as shown by the absence of staining in the parenchyma of the tissues (Figure 4A, B, C). Noteworthy was the finding that stromal compartments (except for the blood vessels) lacked the expression of either gene.

No staining was observed in normal brain and Alzheimer's-diseased brain (Figures 4G, H, I). It is remarkable that ADAM23 and PRSS3 transcripts, whose expression in CNS was revealed by the MTE array and/or reported by RefExA [25], were not detected in normal or diseased brain, except for blood vessels in medulloblastoma or metastatic adenocarcinoma (Figure 4 and data not shown).

Sense probes did not hybridize with any of the tissue sections analyzed (Figures 4D, E, F).

## Discussion

Targeting the process of tumor neovascularization has become a promising anti-cancer strategy. This is particularly evident when vascular targeting agents are combined with conventional chemotherapy and radiotherapy, as demonstrated by recent reports and clinical trials [28]. However, the success of this approach depends on the availability of suitable endothelial markers that can be used to selectively target the tumor vascular compartment or to deliver a ligand-directed effector to the endothelium [7]. Herein, we successfully combined a method to isolate and culture endothelial cells (EC) from human specimens of tumor (TdEC) and non-neoplastic (NdEC) origin with oligonucleotide microarray analysis to compare their gene expression. We first showed that the TdEC transcriptional profile differs from that of NdEC, and, thereafter identified specific molecular features of tumor-derived endothelium.

Our laboratory previously established a procedure [29] to derive EC of high purity from human specimens, their origin assessed by immunohistochemical staining for the three endothelial markers CD31/PECAM-1, VE-cadherin and vWF. Accordingly, microarray results confirmed their expression, but also revealed the expression of many other genes annotated as typically endothelial through SOURCE [30]. These included E-selectin, VEGFR-1, and VEGFR-2, further confirming our previous findings [21]. Despite being less successful with tumors (e.g., a 50% success rate out of 18 ovarian carcinoma specimens) than with non neoplastic (a 90% success rate for adrenal gland), the reproducible yield of EC from human specimens enabled us to explore molecular differences between tumor and normal endothelium.

The comparative transcriptomic analysis of EC from ovarian carcinoma (HOC-EC) and adrenal gland (HA-EC) revealed the higher expression of 158 gene transcripts by HOC-EC. The finding that tumor and normal tissue EC have distinct gene expression patterns concurs with other investigations, which describe genes that are preferentially expressed by endothelium from malignant cancer [12-15]. In these studies, SAGE [12-14] or microarray analyses [15] were executed on EC freshly obtained by immunoselection of *ex vivo *tissue samples. Endothelial cells in culture often lose their tissue-specificity and their specialized properties [31]. Our results, nonetheless, demonstrate that we were able to preserve *in vitro *some of the phenotypes that distinguish the diverse endothelia. Likewise, they confirm our previous findings [21] and agree with other reports [32,33] showing that, despite being in culture, EC isolated from different vascular beds possess characteristic gene expression profiles.

Here we describe for the first time a much higher expression of ADAM23, FAP, GNPNB and PRSS3 in EC derived from six tumor samples than in EC from four normal tissue specimens examined by real-time PCR, whose origin differed from that investigated by microarray analysis. The differences we detected between TdEC and NdEC were maintained whether or not they were exposed to a combination of pro-angiogenic factors that presumably mimic the different environmental conditions under which EC might be embedded *in vivo *(that is, subjected or not to persistent stimulation imposed by the tumor cells, as in the case of blood vessels in adult normal tissues). These results suggest that the transcriptional differences are not culture condition artifacts, but, rather, true differences that distinguish EC of different origin (i.e., tumor vs. normal).

Despite having mainly utilized EC from ovarian cancer and from adrenal glands as the non-neoplastic counterpart, the results hold true for EC isolated from kidney cancer and skin specimens (even though only one EC population could be analyzed). These observations, would seem to indicate that the EC characteristics we observed are not organ-specific, but, rather, exclusive of TdEC. This is in agreement with a recent report [15] that compared EC freshly isolated from ovarian cancer and normal ovary in which several of the genes identified as distinctive of the tumor associated vasculature showed conserved expression across the EC from other tumor types, such as colon [12], brain [13] and breast [14].

Cultures of EC are valuable tools for biochemical and functional analysis, but they do not reflect the physiological situation entirely. All the information should be considered critically from the standpoint of what might occur in blood vessels *in vivo*. To this end, *in situ *hybridization results confirmed ADAM23, GNPNB and PRSS3 expression associated to blood vessels in tumor tissues, but the lack of their expression in cancer cells (as well as in normal tissue).

Overall, these findings support the premise that endothelial markers identified *in vitro *are likely to have *in vivo *relevance, and bear out the validity of our experimental setting designed to reproduce situations that might occur *in vivo*.

ADAM23 belongs to the cellular disintegrins, a family of membrane-anchored proteins that are potential regulators of cell-to-cell and cell-to-matrix interactions [34] and for which a specific binding interaction with alphaVbeta3 integrin has been demonstrated [35]. Our MTE array results showed that ADAM23 is expressed in fetal and adult heart, and also confirmed its previously reported expression by fetal and adult brain [34,35]. However, our *in situ *hybridization finding (lack of gene expression in normal or Alzheimer's-diseased brain) contradicted the latter, which implies that the expression of ADAM23 might indeed be much higher in tumor EC, including blood vessels in medulloblastoma, a highly malignant brain tumor, than in the brain. Further studies, aimed to produce antibodies to ADAM23, are necessary to prove its suitability as a tumor vascular target. Our results also revealed that ADAM23 was not expressed in cancer cells. This observation is consistent with the data available from RefExA and other recent reports [36], which describe hypermethylation of the ADAM23 promoter that silences its expression in breast and pancreatic cell lines and in the corresponding primary tumors. Our evidence of a high expression of ADAM23 by TdEC suggests that this protein might be important for establishing contacts and promoting adhesive functions during the pathological processes leading to the formation of new blood vessels within the tumor. In addition, it is intriguing to speculate that this protein – through its ability to bind alphaVbeta3 – could play some specialized role in the maintenance of vascular functions within the tumor. In keeping with this hypothesis is the fact that alphaVbeta3 is expressed by EC and has been shown to be involved in tumor neo-vascularization [37].

GPNMB is a type I membrane protein identical to the hematopoietic growth factor inducible neurokinin-1 type (HGFIN) [38] sharing similarities with neurokinin-1 (NK1), a receptor for tachykinins and a member of seven transmembrane G protein coupled receptors. HGFIN was shown to interact with Substance P (SP), which has been reported to induce EC proliferation and increase vascular density in an *in vivo *model of inflammation, and these effects could be inhibited by an NK1 receptor antagonist [39]. The action of SP via the NK1 receptor may be a direct effect, since NK1 receptors have previously been localized to endothelium [40], and both SP and selective NK1 agonists were shown to enhance *in vitro *EC proliferation [41]. Substance P *in vivo *also contributes to vascular permeability, plasma extravasation, and edema [42], all features that characterize the abnormal vasculature of tumors. Our evidence of a high expression of GPNMB by TdEC suggests that GPNMB may be a tumor stroma receptor for SP, involved in tumor angiogenic events and in determining the distinctive features of tumor vasculature. Corroborating this premise are data suggesting that, in addition to NK1 receptors, other mechanisms contribute in mediating *in vivo *SP effects on EC [39].

The PRSS3 gene encodes for a trypsinogen whose functions have not yet been fully elucidated [43]. PRSS3 transcript, whose expression in CNS is reported by RefExA, was not detected in normal or Alzheimer's-diseased brain, except for blood vessels in a brain metastasis. This finding might mean that the expression of PRSS3 is indeed characteristic of tumor EC, as seen for ADAM23. Our search in the RefExA and SOURCE databases revealed that PRSS3 was not expressed by tumor cells, with the exception of colon carcinoma. It seems very plausible that PRSS3 produced by TdEC may contribute to tumor angiogenesis, invasion and metastasis through its peptidase activity. This notion upholds evidence that the migration and invasion of EC in the tumor tissue requires the activation of pro-enzymes (such as pro-metalloproteinases) and protein precursors [44]. To this end, it is noteworthy that other peptidases (including urokinase-plasminogen activator [uPA] and FAP) were among the genes we found to be highly expressed by tumor-derived endothelium. In particular, uPA is capable of initiating the process of extracellular matrix degradation through the activation of plasminogen and matrix metalloproteases; in addition, it has been shown to be required for the migration of EC during the process of angiogenesis [45]. It is interesting that the expression of the uPA receptor (uPAR) was also seen to be higher in HOC-EC than in HA-EC (C. Ghilardi unpublished observation), a finding that would implicate the uPA-uPAR system in tumor driven angiogenesis.

Our results show that the cell surface protease FAP was also expressed at significantly higher levels by TdEC than by normal EC. FAP was originally reported to be an antigen recognized by F19 antibody in cultured fibroblasts [27]. Accordingly, its expression in our tests was found to be much higher in cultured fibroblasts (HuFb and MALME3) and in smooth muscle cells (UASMC). Nonetheless, while normal adult tissues are generally FAP-negative, its expression is detectable in the stroma of over 90% of carcinomas [27]. Such behavior suggested its suitability for stromal targeting and/or cancer detection and therapy, and in recent years has underpinned the development of a humanized anti-FAP antibody (sibrotuzumab) and the implementation of clinical trials [46]. Our results uphold the notion that FAP is a valid target for therapy, be it as stromal or as vascular marker. In keeping with our results, the *in vivo *expression of FAP on blood vessels was recently demonstrated by Ghersi et al. in invasive breast ductal carcinoma [47].

Many of the transcripts we found to be expressed at a high level by EC from neoplastic tissues were previously reported as being involved in tumor angiogenesis or expressed by tumor EC. In this regard, it is worth listing the various types of matrix proteins that we detected, namely: collagens alpha-2 type I, alpha-1 type III, V, XIII and XV, glypican-1, laminin alpha3, and tenascin C. Our results confirm similar findings from other laboratories [12,13,15] and denote a remodeling of the extracellular matrix known to promote blood vessel sprouting and growth. In keeping with the experimental data, and as an example, tenascin C was shown to be expressed *in vivo *and to colocalize with microvessels in the stroma of non-small cell lung cancer [48].

The expression of cell adhesion proteins was also altered in TdEC. This may be explained by the fact that migrating and proliferating EC must "loosen up", that is, detach and then create new interactions. Our results implicate CD44 and cadherins in this process, and confirm previous reports showing N-cadherin over-expression on proliferative state EC [49], and enhanced expression of CD44 on the vasculature of solid tumors compared to normal tissue [50]. Noteworthy is the finding that CD44 expression was up-regulated when the TdEC were exposed to angiogenic growth factors (C. Ghilardi unpublished observations), in accordance with previous results showing that FGF-2 and VEGF stimulate CD44 expression in EC [50].

Interestingly, ABCA2 and ABCB1 (MDR-1/p-glycoprotein 1) were also more abundantly expressed by TdEC, reflecting the previously reported higher expression of p-glycoprotein 1 in EC from glioma than from normal brain [51]. The ATP-binding cassette transporters (ABC) are responsible for the efflux of chemotherapeutic agents in cancer cells [52]. These data suggest that the multidrug resistance phenomenon in tumors may be due to both EC and tumor cells.

Molecules such as SMAD7, BAMBI and BCL2 are listed among the transcripts that are more highly expressed by HOC-EC than by HA-EC. SMAD7 and BAMBI interfere with the TGFβ pathway and may potentially block the anti-proliferative action of TGFβ [53]. Their expression may induce an enhanced proliferation of the TdEC, which is one of the steps of the angiogenic process. BCL2 is one of the most widely recognized anti-apoptotic factors, whose upregulation enhances EC survival and intratumoral angiogenesis, thus promoting tumor growth [54].

Emerging studies suggest that the vascular and the nervous systems share the same molecular signals in their development. The involvement of netrins, semaphorins, Robo/Slit and VEGF families in both angiogenesis and neurogenesis has been demonstrated in recent years [24]. In keeping with these findings, our results revealed that TdEC express numerous molecules that are associated with the CNS or are implicated in brain differentiation and axon guidance. In particular, ADORA2B was identified as a novel netrin-1 receptor and reported to mediate axon outgrowth [55]. In a similar way – and considering that netrin-1 is pro-angiogenic [56] – our results allow speculatation that ADORA2B could mediate the sprouting of vessels during tumor neovascularization.

## Conclusion

Targeting angiogenesis and vasculature represents a promising option to control tumor growth and dissemination. To exploit this opportunity, however, selective targets in tumor associated vasculature need to be identified.

We describe a model consisting of *in vitro *cultured endothelial cells isolated from human normal and neoplastic specimens that maintained their respective molecular phenotype and enabled us to determine molecular differences between tumor and normal tissue derived EC. Specifically, we identified four transcripts whose expression is much higher in endothelial cells from tumors than from normal tissue. The *in vivo *expression in cancer-associated blood vessels was confirmed by *in situ *hybridization of human specimens.

This work evinces some of the distinctive features of tumor-derived endothelium, and identifies potential markers that may provide the groundwork for novel therapeutic strategies. Future studies are needed to clarify the functions and roles of these molecules in tumor progression and vascularization.

## Methods

### Isolation, culture and characterization of endothelial cells (EC)

The method for isolation of EC has been described in detail by [29,21]. Briefly, tissue specimens were digested by type I collagenase (EC 3.4.24.3 – clostridiopeptidase A, Sigma-Aldrich, St. Louis MO, USA – Milan, Italy) and the suspension plated. Six to 10 days later, the EC were positively purified using anti-CD31 antibody-coated magnetic beads, seeded and cultured as detailed in the supplementary information [see Additional file 1].

Human umbilical vein EC (HUVEC) were obtained following the procedure described by Jaffe [57] and cultured as detailed in [58].

To confirm their endothelial origin, EC cultures were analyzed by immunohistochemical methods to evaluate the expression of von Willebrand Factor (vWF), platelet-endothelial cell adhesion molecule-1 (CD31/PECAM-1), Vascular E-cadherin (VE-cadherin), and alpha-smooth muscle Actin (alpha-SMA), and by fluorescence activated cell sorter (FACS) to evaluate low-density lipoprotein (LDL) uptake and CD31/PECAM-1 antigen expression, as previously described [21].

### Cell cultures

HT29 colon carcinoma cells (ATCC-American Type Culture Collection) were grown in minimum essential medium (MEM) supplemented with 10% Fetal Bovine Serum (FBS).

1A9 (a subclone of the A2780 cell line [59]) and SKOV3 human ovarian carcinoma cells, MDA-MB-231 human breast carcinoma cells, and HuFb and Malme-3 human fibroblasts (all from ATCC) were grown in RPMI-1640 medium plus 10% FBS.

Umbilical artery smooth muscle cells (UASMC) were purchased from Clonetics (Clonetics-BioWhittaker, Walkersville MD, USA – Caravaggio BG, Italy) and cultured following the manufacturer's protocol in SmBM Growth Medium.

### RNA isolation

Total RNA was isolated from cultured cells using Trizol^® ^(GIBCO-Invitrogen) or RNeasy Mini Kit (Qiagen, Maryland, USA) following the manufacturer's protocols. Potential genomic DNA contaminations were removed by DNAse (Ambion, Austin, USA) treatment followed by RNA CleanUp with RNeasy Mini Kit. The purity and integrity of the RNA was checked by gel electrophoresis, and concentration was determined spettrophotometrically.

### Genome wide analysis of gene expression by microarray

#### Transcriptional difference: HOC-EC vs. HA-EC

We are aware that culture conditions alter gene expression. To this end, all the EC under investigation were analyzed under two different conditions: either seeded onto type I collagen coated tissue culture plastic (BioCoat-Becton Dickinson, Bedford MA, USA) in endothelial cell basal medium (EBM – Clonetics-BioWhittaker, Walkersville MD, USA) plus 5% FBS or onto BioCoat plasticware additionally coated with 1 μg/cm^2 ^fibronectin (Plasma Fibronectin, BD-Biosciences, Bedford MA, USA) in EBM plus 5% FBS supplemented with and 10 ng/ml Epidermal Growth Factor (EGF), 10 ng/ml Vascular Endothelial Growth Factor (VEGF), 2 ng/ml Fibroblast Growth Factor (FGF-2) (all from R&D System, Minneapolis MN, USA); from here on respectively called "unexposed" or "exposed" to a tumor/angiogenic environment.

To maximize the likelihood of detecting the intrinsic differences between HOC-EC and HA-EC by minimizing the influence of the artificial environment upon them, we opted to select only those genes that were differentially expressed regardless of the culture conditions.

##### Microarray analysis

GeneChip^® ^Human Genome U95A Arrays (Affymetrix UK Ltd, High Wycombe, UK) that monitor the expression of approximately 12,000 genes were utilized to analyze the transcriptional profiles of 3 independent HOC-EC together with 2 HA-EC populations, under both "exposed" and "unexposed" culture conditions. Raw Affymetrix data were then processed for each of the 10 GeneChip^® ^arrays.

##### Data analysis

For each probeset, the mean of the average difference values for the two "exposed" HA-EC samples was calculated only if both were greater than 20 arbitrary units. Similarly, the mean of at least two average difference values for the three "exposed" HOC-EC samples was calculated only if each of them was greater than 20 arbitrary units. The same was done for the "unexposed" samples. Then, the log2 of the HOC-EC versus HA-EC ratios was calculated only for the probesets having both means available. Probesets with log-ratios greater than 1.3 (corresponding to a fold change of 2.5) for both "exposed" and "unexposed" samples were considered. Such a threshold was obtained by fitting the distributions of the "exposed" and "unexposed" log2-ratios with a normal distribution and calculating upper bound of the 99% confidence interval.

##### Gene annotation

the probesets expressed to a greater extent in HOC-EC than in HA-EC were divided into functional classes using the publicly available Est Annotation and the Keyword Clustering Machines [60] and were then manually checked.

#### Typical endothelial genes: HOC-EC and HA-EC

Endothelial markers represented on the Affymetrix GeneChip^® ^were found by keyword search within the complete probeset list annotated using the publicly available SOURCE batch search [30,61]. The mean of average difference values for each transcript in HOC-EC and HA-EC was calculated. Genes with the mean value greater than 500 were included in the list of expressed endothelial markers shown in Table 2.

Additional information on gene expression was obtained from the publicly available Reference database for gene Expression Analysis (RefExA) of the Laboratory for System Biology and Medicine (LSBM) (RCAST, University of Tokyo) [25].

### Quantitative real-time PCR

Two micrograms of total cellular RNA were reverse transcribed (RT) using the Taqman Reverse Transcription Reagents and Random Hexamer primers (Applied Biosystems, Foster City, CA, USA) according to the manufacturer's protocols. To check for DNA contamination, a control RT reaction was set up for each sample without the addition of reverse transcriptase.

Quantitative real-time PCR was performed in 25 μl-reactions on the GeneAmp 5700 Sequence Detection System (Applied Biosystems) using either SYBR Green or TaqMan chemistry. Primer sets for PCR amplifications are listed in supplementary table 1 [see Additional file 1] and were designed using the Primer Express 1.5 software (Applied Biosystems) and synthesized by Gibco-Life Technologies. Primer concentration was 200 nM for all SYBR Green assays, except for 18s rRNA assay (50 nM); dissociation curves were routinely performed. For transcripts analyzed with TaqMan chemistry, specific Assay-on-Demands were purchased from Applied Biosystems [see supplementary table 1 in Additional file 1].

All assays were performed using duplicate samples of each cDNA synthesis. Reverse transcription reactions and real-time PCR analyses were carried out at least twice for each gene transcript to determine consistency of results.

Gene expression of all transcripts was normalized to the endogenous control gene human 18s rRNA and for each population the ΔCt value was calculated (ΔCt = Ct_targetgene _- Ct _18s_). Statistical analysis with Student's t-test (unpaired) on ΔCt values was performed to compare the expression levels of target genes in EC samples from normal and neoplastic tissues.

Fold differences were calculated according to the comparative ΔΔCt method (GeneAmp 5700 SDS User's Bulletin; Applied Biosystems).

### Northern Blot

For the analysis of tissue distribution, the MTE Array 2 (Clontech, Palo Alto CA, USA) was hybridized following manufacturer's instructions. Briefly, the filter was pre-hybridized for 30 min at 65°C with ExpressHyb (Clontech). Hybridization was carried out O.N. at 65°C in ExpressHyb containing 130 μg/ml fish sperm DNA, 6 μg/ml Cot -1 DNA and the ^32^P-labeled cDNA probe. Filter was washed with SSC2x/SDS0.1% at 65°C, followed by washes at 55°C with SSC0.1x/SDS 0.5%. Because the panel of human mRNAs loaded onto the MTE Arrays in dot-blot format are normalized using eight different housekeeping genes, the hybridization results can be attributed to actual differences in target mRNA abundance.

Densitometric analysis of the dots was performed with Gel-Pro Analyzer Software (Media Cybernetics, Silver Spring, MD). Optical Density (OD) values are reported in Figure 3.

cDNA probe for ADAM23 corresponding to nt 2241–2743 of GeneBank:NM_003812, and cDNA probe for GPNMB corresponding to nt 416–984 of GeneBank:NM_002510 were PCR amplified and gel purified.

### *In situ *hybridization

*In situ *hybridization experiments were carried out with a mixture of specific biotin-labeled oligonucleotide antisense (or sense) probes for ADAM23, GPNMB and PRSS3 (Metabion International AG, Martinsried, Germany) listed in the supplementary Table 2 [see Additional file 1]. Human cancer specimens, such as adenocarcinoma brain metastasis, angioma from a Von Hippel Lindau disease patient, Ewing sarcoma, and medulloblastoma, were analyzed along with normal brain and Alzheimer's-diseased brain.

After removal of paraffin, the tissue sections were rehydrated and heat-treated in 0.1 M sodium citrate (pH = 6), washed for 5 min with water and then with 2× SSC, and air dried. Sections were allowed to hybridize overnight at room temperature in 15% formamide, 5× SSC, 10% dextran sulphate, 1× Denhardt's solution (0.02% Ficoll400, 0.02% polyvinylpyrrolidone, 0.02% bovine serum albumin) and 400 μg/ml fish sperm DNA. Probe concentration was 10 nM for each oligonucleotide (corresponding to 10 fmol/μl). Approximately 100 μl of hybridization solution was applied to each slide.

After hybridization, sections were washed for 15 min in 2× SSC and for 10 min in water at room temperature. They were rinsed three times in PBS/0.1% Triton, and incubated for 20 min with blocking buffer (PBS/0.1% Triton, 5% FCS). Bound probes were detected using a mouse anti-biotin antibody (DakoCytomation, Glostrup, Denmark). Amplification of the signal was achieved with the Vectastain elite ABC Kit (Vector Laboratories, Burlingame, CA) according to the manufacturer's protocol. Colorimetric detection was completed with the AEC Substrate kit (Vector Laboratories). Finally, the tissue sections were counterstained with hematoxylin and embedded on an aqueous mounting medium.

### Ethical consent

Endothelial cells have been isolated from biopsies collected at the San Gerardo Hospital (Monza, Italy) from patients undergoing therapeutic surgery from September 1998 to March 2004. The collection and use of the tissue samples was approved by the Local Scientific Ethical Committee in compliance with the principles enunciated in the Helsinki Declaration [62]. Tissue samples (including tumors) used for in situ hybridization were acquired from the Oxford based Thomas Willis brain bank. The collection of the tissues was done in accordance with the requirements of the Human Tissue Act, with full informed consent of participants and relatives. Ethical approval for research to be conducted on the tissues was granted by COREC (No: 1656).

## Abbreviations

ADAM23: a disintegrin and metalloproteinase domain 23; Alpha-SMA: alpha-smooth muscle actin; CD31/PECAM-1: platelet-endothelial cell adhesion molecule-1; CNS: central nervous system; EBM: endothelial cell basal medium; EC: endothelial cells; EGF: epidermal growth factor; FAP: fibroblast activation protein; FBS: fetal bovine serum; FDA: food and drug administration; FGF-2: fibroblast growth factor-2 (basic); GPNMB: glycoprotein nmb; HA-EC: human adrenal gland endothelial cells; HKC-EC: human kidney carcinoma endothelial cells; HOC-EC: human ovarian carcinoma endothelial cells; HSk-EC: human skin derived endothelial cells; HUVEC: human umbilical vein endothelial cells; LDL: low density lipoproteins; MEM: minimum essential medium; MTE Array: multiple tissue expression array; NdEC: non-neoplastic derived endothelial cells; NK1: neurokinin-1; OD, optical density; PAR-1: protease activated receptor-1; PCR: polymerase chain reaction; PRSS3: serine protease 3; SAGE: serial analysis of gene expression; SCGE: sarcoglycan epsilon; SmBM: smooth muscle basal medium; SP: substance P; TdEC: tumor derived endothelial cells; uPA: urokinase-plasminogen activator; uPAR: urokinase-plasminogen activator receptor; VEGF: vascular endothelial growth factor; VE-cadherin: vascular endothelial-cadherin; VWF: von Willebrand factor.

## Authors' contributions

CG carried out the gene expression studies and drafted the manuscript. GC performed the mathematical and statistical analysis. RD participated in endothelial cell isolation. ZN participated in *in situ *hybridization experiments. RG participated in the design of the study and preparation of the manuscript. MRB conceived of the study and participated in its design and coordination and help to draft the manuscript. All authors read and approved the final manuscript.

## Supplementary Material

Additional file 1 **CGhilardi_Supplementary information**. This file contains: Isolation and culture of endothelial cells, Supplementary table 1 – Primers and Assay-on-Demand used in quantitative real-time PCR-, Supplementary table 2 – Biotin-labelled oligonucleotides used for In Situ Hybridization -. Click here for file

## References

[B1] Ferrara N, Kerbel RS (2005). Angiogenesis as a therapeutic target. Nature.

[B2] Hurwitz H, Fehrenbacher L, Novotny W, Cartwright T, Hainsworth J, Heim W, Berlin J, Baron A, Griffing S, Holmgren E, Ferrara N, Fyfe G, Rogers B, Ross R, Kabbinavar F (2004). Bevacizumab plus irinotecan, fluorouracil, and leucovorin for metastatic colorectal cancer. N Engl J Med.

[B3] Folkman J (1995). Angiogenesis in cancer, vascular, rheumatoid and other disease. Nat Med.

[B4] Manenti L, Paganoni P, Floriani I, Landoni F, Torri V, Buda A, Taraboletti G, Labianca R, Belotti D, Giavazzi R (2003). Expression levels of vascular endothelial growth factor, matrix metalloproteinases 2 and 9 and tissue inhibitor of metalloproteinases 1 and 2 in the plasma of patients with ovarian carcinoma. Eur J Cancer.

[B5] O'Reilly MS, Holmgren L, Shing Y, Chen C, Rosenthal RA, Moses M, Lane WS, Cao Y, Sage EH, Folkman J (1994). Angiostatin: a novel angiogenesis inhibitor that mediates the suppression of metastases by a Lewis lung carcinoma. Cell.

[B6] Papetti M, Herman IM (2002). Mechanisms of normal and tumor-derived angiogenesis. Am J Physiol Cell Physiol.

[B7] Neri D, Bicknell R (2005). Tumour vascular targeting. Nat Rev Cancer.

[B8] Pasqualini R, Ruoslahti E (1996). Organ targeting In vivo using phage display peptide libraries. Nature.

[B9] Hoffman JA, Giraudo E, Singh M, Zhang L, Inoue M, Porkka K, Hanahan D, Ruoslahti E (2003). Progressive vascular changes in a transgenic mouse model of squamous cell carcinoma. Cancer Cell.

[B10] Oh P, Li Y, Yu J, Durr E, Krasinska KM, Carver LA, Testa JE, Schnitzer JE (2004). Subtractive proteomic mapping of the endothelial surface in lung and solid tumours for tissue-specific therapy. Nature.

[B11] Rybak JN, Ettorre A, Kaissling B, Giavazzi R, Neri D, Elia G (2005). In vivo protein biotinylation for identification of organ-specific antigens accessible from the vasculature. Nat Methods.

[B12] St Croix B, Rago C, Velculescu V, Traverso G, Romans KE, Montgomery E, Lal A, Riggins GJ, Lengauer C, Vogelstein B, Kinzler KW (2000). Genes expressed in human tumor endothelium. Science.

[B13] Madden SL, Cook BP, Nacht M, Weber WD, Callahan MR, Jiang Y, Dufault MR, Zhang X, Zhang W, Walter-Yohrling J, Rouleau C, Akmaev VR, Wang CJ, Cao X, St Martin TB, Roberts BL, Teicher BA, Klinger KW, Stan RV, Lucey B, Carson-Walter EB, Laterra J, Walter KA (2004). Vascular gene expression in nonneoplastic and malignant brain. Am J Pathol.

[B14] Parker BS, Argani P, Cook BP, Liangfeng H, Chartrand SD, Zhang M, Saha S, Bardelli A, Jiang Y, St Martin TB, Nacht M, Teicher BA, Klinger KW, Sukumar S, Madden SL (2004). Alterations in vascular gene expression in invasive breast carcinoma. Cancer Res.

[B15] Lu C, Bonome T, Li Y, Kamat AA, Han LY, Schmandt R, Coleman RL, Gershenson DM, Jaffe RB, Birrer MJ, Sood AK (2007). Gene Alterations Identified by Expression Profiling in Tumor-Associated Endothelial Cells from Invasive Ovarian Carcinoma. Cancer Res.

[B16] Kahn J, Mehraban F, Ingle G, Xin X, Bryant JE, Vehar G, Schoenfeld J, Grimaldi CJ, Peale F, Draksharapu A, Lewin DA, Gerritsen ME (2000). Gene expression profiling in an in vitro model of angiogenesis. Am J Pathol.

[B17] Roland I, Minet E, Ernest I, Pascal T, Michel G, Remacle J, Michiels C (2000). Identification of hypoxia-responsive messengers expressed in human microvascular endothelial cells using differential display RT-PCR. Eur J Biochem.

[B18] Wang JL, Liu YH, Lee MC, Nguyen TM, Lee C, Kim A, Nguyen M (2000). Identification of tumor angiogenesis-related genes by subtractive hybridization. Microvasc Res.

[B19] Khodarev NN, Yu J, Labay E, Darga T, Brown CK, Mauceri HJ, Yassari R, Gupta N, Weichselbaum RR (2003). Tumour-endothelium interactions in co-culture: coordinated changes of gene expression profiles and phenotypic properties of endothelial cells. J Cell Sci.

[B20] Scheurer SB, Rybak JN, Rosli C, Neri D, Elia G (2004). Modulation of gene expression by hypoxia in human umbilical cord vein endothelial cells: A transcriptomic and proteomic study. Proteomics.

[B21] Alessandri G, Chirivi RG, Fiorentini S, Dossi R, Bonardelli S, Giulini SM, Zanetta G, Landoni F, Graziotti PP, Turano A, Caruso A, Zardi L, Giavazzi R, Bani MR (1999). Phenotypic and functional characteristics of tumour-derived microvascular endothelial cells. Clin Exp Metastasis.

[B22] Ricciardelli C, Rodgers RJ (2006). Extracellular matrix of ovarian tumors. Semin Reprod Med.

[B23] van Hinsbergh VW, Engelse MA, Quax PH (2006). Pericellular proteases in angiogenesis and vasculogenesis. Arterioscler Thromb Vasc Biol.

[B24] Carmeliet P (2003). Blood vessels and nerves: common signals, pathways and diseases. Nat Rev Genet.

[B25] RefExA Reference database for gene Expression Analysis (RefExA). http://www.lsbm.org.

[B26] Szmola R, Kukor Z, Sahin-Toth M (2003). Human mesotrypsin is a unique digestive protease specialized for the degradation of trypsin inhibitors. J Biol Chem.

[B27] Garin-Chesa P, Old LJ, Rettig WJ (1990). Cell surface glycoprotein of reactive stromal fibroblasts as a potential antibody target in human epithelial cancers. Proc Natl Acad Sci U S A.

[B28] Horsman MR, Siemann DW (2006). Pathophysiologic effects of vascular-targeting agents and the implications for combination with conventional therapies. Cancer Res.

[B29] Alessandri G, Chirivi RG, Castellani P, Nicolo G, Giavazzi R, Zardi L (1998). Isolation and characterization of human tumor-derived capillary endothelial cells: role of oncofetal fibronectin. Lab Invest.

[B30] Diehn M, Sherlock G, Binkley G, Jin H, Matese JC, Hernandez-Boussard T, Rees CA, Cherry JM, Botstein D, Brown PO, Alizadeh AA (2003). SOURCE: a unified genomic resource of functional annotations, ontologies, and gene expression data. Nucleic Acids Res.

[B31] Garlanda C, Dejana E (1997). Heterogeneity of endothelial cells. Specific markers. Arterioscler Thromb Vasc Biol.

[B32] Chi JT, Chang HY, Haraldsen G, Jahnsen FL, Troyanskaya OG, Chang DS, Wang Z, Rockson SG, van de Rijn M, Botstein D, Brown PO (2003). Endothelial cell diversity revealed by global expression profiling. Proc Natl Acad Sci U S A.

[B33] Ho M, Yang E, Matcuk G, Deng D, Sampas N, Tsalenko A, Tabibiazar R, Zhang Y, Chen M, Talbi S, Ho YD, Wang J, Tsao PS, Ben-Dor A, Yakhini Z, Bruhn L, Quertermous T (2003). Identification of endothelial cell genes by combined database mining and microarray analysis. Physiol Genomics.

[B34] Sagane K, Ohya Y, Hasegawa Y, Tanaka I (1998). Metalloproteinase-like, disintegrin-like, cysteine-rich proteins MDC2 and MDC3: novel human cellular disintegrins highly expressed in the brain. Biochem J.

[B35] Cal S, Freije JM, Lopez JM, Takada Y, Lopez-Otin C (2000). ADAM 23/MDC3, a human disintegrin that promotes cell adhesion via interaction with the alphavbeta3 integrin through an RGD-independent mechanism. Mol Biol Cell.

[B36] Hagihara A, Miyamoto K, Furuta J, Hiraoka N, Wakazono K, Seki S, Fukushima S, Tsao MS, Sugimura T, Ushijima T (2004). Identification of 27 5' CpG islands aberrantly methylated and 13 genes silenced in human pancreatic cancers. Oncogene.

[B37] Brooks PC, Clark RA, Cheresh DA (1994). Requirement of vascular integrin alpha v beta 3 for angiogenesis. Science.

[B38] Bandari PS, Qian J, Yehia G, Joshi DD, Maloof PB, Potian J, Oh HS, Gascon P, Harrison JS, Rameshwar P (2003). Hematopoietic growth factor inducible neurokinin-1 type: a transmembrane protein that is similar to neurokinin 1 interacts with substance P. Regul Pept.

[B39] Seegers HC, Hood VC, Kidd BL, Cruwys SC, Walsh DA (2003). Enhancement of angiogenesis by endogenous substance P release and neurokinin-1 receptors during neurogenic inflammation. J Pharmacol Exp Ther.

[B40] Walsh DA, Salmon M, Mapp PI, Wharton J, Garrett N, Blake DR, Polak JM (1993). Microvascular substance P binding to normal and inflamed rat and human synovium. J Pharmacol Exp Ther.

[B41] Ziche M, Morbidelli L, Pacini M, Geppetti P, Alessandri G, Maggi CA (1990). Substance P stimulates neovascularization in vivo and proliferation of cultured endothelial cells. Microvasc Res.

[B42] Xu XJ, Dalsgaard CJ, Maggi CA, Wiesenfeld-Hallin Z (1992). NK-1, but not NK-2, tachykinin receptors mediate plasma extravasation induced by antidromic C-fiber stimulation in rat hindpaw: demonstrated with the NK-1 antagonist CP-96,345 and the NK-2 antagonist Men 10207. Neurosci Lett.

[B43] Nyaruhucha CN, Kito M, Fukuoka SI (1997). Identification and expression of the cDNA-encoding human mesotrypsin(ogen), an isoform of trypsin with inhibitor resistance. J Biol Chem.

[B44] Heissig B, Hattori K, Friedrich M, Rafii S, Werb Z (2003). Angiogenesis: vascular remodeling of the extracellular matrix involves metalloproteinases. Curr Opin Hematol.

[B45] Binder BR, Mihaly J, Prager GW (2007). uPAR-uPA-PAI-1 interactions and signaling: a vascular biologist's view. Thromb Haemost.

[B46] Scott AM, Wiseman G, Welt S, Adjei A, Lee FT, Hopkins W, Divgi CR, Hanson LH, Mitchell P, Gansen DN, Larson SM, Ingle JN, Hoffman EW, Tanswell P, Ritter G, Cohen LS, Bette P, Arvay L, Amelsberg A, Vlock D, Rettig WJ, Old LJ (2003). A Phase I dose-escalation study of sibrotuzumab in patients with advanced or metastatic fibroblast activation protein-positive cancer. Clin Cancer Res.

[B47] Ghersi G, Zhao Q, Salamone M, Yeh Y, Zucker S, Chen WT (2006). The protease complex consisting of dipeptidyl peptidase IV and seprase plays a role in the migration and invasion of human endothelial cells in collagenous matrices. Cancer Res.

[B48] Ishiwata T, Takahashi K, Shimanuki Y, Ohashi R, Cui R, Takahashi F, Shimizu K, Miura K, Fukuchi Y (2005). Serum tenascin-C as a potential predictive marker of angiogenesis in non-small cell lung cancer. Anticancer Res.

[B49] Zhang HT, Gorn M, Smith K, Graham AP, Lau KK, Bicknell R (1999). Transcriptional profiling of human microvascular endothelial cells in the proliferative and quiescent state using cDNA arrays. Angiogenesis.

[B50] Griffioen AW, Coenen MJ, Damen CA, Hellwig SM, van Weering DH, Vooys W, Blijham GH, Groenewegen G (1997). CD44 is involved in tumor angiogenesis; an activation antigen on human endothelial cells. Blood.

[B51] Regina A, Demeule M, Berube A, Moumdjian R, Berthelet F, Beliveau R (2003). Differences in multidrug resistance phenotype and matrix metalloproteinases activity between endothelial cells from normal brain and glioma. J Neurochem.

[B52] Gillet JP, Efferth T, Remacle J (2007). Chemotherapy-induced resistance by ATP-binding cassette transporter genes. Biochim Biophys Acta.

[B53] Funaki T, Nakao A, Ebihara N, Setoguchi Y, Fukuchi Y, Okumura K, Ra C, Ogawa H, Kanai A (2003). Smad7 suppresses the inhibitory effect of TGF-beta2 on corneal endothelial cell proliferation and accelerates corneal endothelial wound closure in vitro. Cornea.

[B54] Nor JE, Christensen J, Liu J, Peters M, Mooney DJ, Strieter RM, Polverini PJ (2001). Up-Regulation of Bcl-2 in Microvascular Endothelial Cells Enhances Intratumoral Angiogenesis and Accelerates Tumor Growth. Cancer Res.

[B55] Corset V, Nguyen-Ba-Charvet KT, Forcet C, Moyse E, Chedotal A, Mehlen P (2000). Netrin-1-mediated axon outgrowth and cAMP production requires interaction with adenosine A2b receptor. Nature.

[B56] Park KW, Crouse D, Lee M, Karnik SK, Sorensen LK, Murphy KJ, Kuo CJ, Li DY (2004). The axonal attractant Netrin-1 is an angiogenic factor. PNAS.

[B57] Jaffe EA, Nachman RL, Becker CG, Minick CR (1973). Culture of human endothelial cells derived from umbilical veins. Identification by morphologic and immunologic criteria. J Clin Invest.

[B58] Taraboletti G, Sonzogni L, Vergani V, Hosseini G, Ceruti R, Ghilardi C, Bastone A, Toschi E, Borsotti P, Scanziani E, Giavazzi R, Pepper MS, Stetler-Stevenson WG, Bani MR (2000). Posttranscriptional stimulation of endothelial cell matrix metalloproteinases 2 and 1 by endothelioma cells. Exp Cell Res.

[B59] Bani MR, Nicoletti MI, Alkharouf NW, Ghilardi C, Petersen D, Erba E, Sausville EA, Liu ET, Giavazzi R (2004). Gene expression correlating with response to paclitaxel in ovarian carcinoma xenografts. Mol Cancer Ther.

[B60] ESTannotation Keyword Clustering Machines. http://bio.ifom-firc.it.

[B61] SOURCE SOURCE. http://smd-www.stanford.edu/cgi-bin/source/sourceSearch.

[B62] Declaration H Helsinky Declaration. http://www.wma.net/e/policy/b3.htm.

