# Supplementary material for: Identification of novel vascular markers through gene expression profiling of tumor-derived endothelium
Source: BMC Genomics. 2008 Apr 30;9:201. doi: 10.1186/1471-2164-9-201 (PMC2410137; doi:10.1186/1471-2164-9-201)
Supplement: Additional file 1 — CGhilardi_Supplementary information. This file contains: Isolation and culture of endothelial cells, Supplementary table 1 – Primers and Assay-on-Demand used in quantitative real-time PCR-, Supplementary table 2 – Biotin-labelled oligonucleotides used for In Situ Hybridization -. [file 1471-2164-9-201-S1.doc]

**Supplementary information**

**Isolation and culture of endothelial cells:**

The methodology utilized for isolating endothelial cells from normal and neoplastic human specimens was optimized in our lab and it has been described in [1, 2].

Human tissue fragments, after being repeatedly washed in Phosphate Buffer Saline (PBS) supplemented with amphotericin B (7.5 μg/ml), penicillin (300 U/ml) and streptomycin (300 μg/ml), and gentamicin (150 µg/ml), all from GIBCO-Invitrogen (Carlsbad CA, USA - S.Giuliano Milanese MI, Italy), were finely minced with scissors and then enzymatically digested for 30-60 min at 37°C by shaking incubation in M199 medium (Biochrom AG, Berlin, Germany) containing 0.25% w/v of type I collagenase (EC 3.4.24.3 - clostridiopeptidase A, Sigma-Aldrich, St. Louis MO, USA - Milan, Italy) and 0.25% w/v Bovine Serum Albumin (BSA-Sigma) plus amphotericin B (5 μg/ml), penicillin (200 U/ml) and streptomycin (200 μg/ml).

The cell suspensions were then washed in M199 plus 10% Fetal Bovine Serum (FBS), passed through 100µm, 50µm and 20µm pore size filters (ConsulTS Turin, Italy). This procedure was performed to allow an easy removal of unwanted cellular macroaggregates. The flow through was centrifuged, and the pellet resuspended in *EBM growth medium*, consisting of endothelial cell basal medium (EBM-Clonetics-BioWhittaker, Walkersville MD, USA - Caravaggio BG, Italy) supplemented with 10% FBS, 15 U/ml heparin (Parke-Davis, Milan, Italy), 1 μg/ml hydrocortisone (Sigma), 10 ng/ml human recombinant Epidermal Growth Factor (EGF, R&D System, Minneapolis MN, USA), 50 μg/ml endothelial cell growth supplement (ECGS, Sigma) additioned of antibiotics (amphotericin B 2.5 μg/ml, penicillin 100 U/ml and streptomycin 100 μg/ml). If the cell suspension was obtained from normal tissue it was plated onto tissue culture dishes coated with Collagen I (BioCoat, Becton Dickinson, Bedford, MA, USA). If the cell suspension was obtained from tumor specimens it was plated onto BioCoat tissue culture plastic additionally coated with 1 μg/cm^2^ of FN-WI38VA (Fibronectin from WI-38VAI3 fibroblasts purified from conditioned supernatant; [3] and the  *EBM growth medium* was supplemented with 5 ng/ml of vascular endothelial growth factor (VEGF, R&D System).

The primary cultures were later (6-10 days) harvested by trypsinisation (trypsin 0.25% w/v) washed and resuspended 1x10^6^/ml in PBS plus 1% BSA. The cells were gently mixed for 30 min a 4°C with anti-CD31 monoclonal antibodies (clone 5FY/9, obtained through the courtesy of M.G. Lampugnani, IFOM, FIRC Institute of Molecular Oncology, Milan, Italy) covalently bound to magnetic beads (Dynabeads M450 Rat Anti-Mouse IgG - DYNAL, Oslo, Norway) [cells:beads= 1:3]. Endothelial cells were positively purified by using a magnet (Magnetic Particles Concentrator-DYNAL) and then seeded (1.5-2x10^4^ cells/cm^2^) as described above. This was assumed as the first *in vitro* passage.

From the second *in vitro* passage on, fibronectin from plasma (Plasma Fibronectin, BD-Biosciences, Bedford MA, USA) was used in substitution of FN-WI38VA as substratum for tumor-derived endothelial cells. All the endothelial cells were used between 3-5 *in vitro* passages.

**References**

1. Alessandri G, Chirivi RG, Castellani P, Nicolo G, Giavazzi R, Zardi L: Isolation and characterization of human tumor-derived capillary endothelial cells: role of oncofetal fibronectin. *Lab Invest* 1998, 78(1):127-128.

2. Alessandri G, Chirivi RG, Fiorentini S, Dossi R, Bonardelli S, Giulini SM, Zanetta G, Landoni F, Graziotti PP, Turano A *et al*: Phenotypic and functional characteristics of tumour-derived microvascular endothelial cells. *Clin Exp Metastasis* 1999, 17(8):655-662.

3. Zardi L, Carnemolla B, Siri A, Petersen TE, Paolella G, Sebastio G, Baralle FE: Transformed human cells produce a new fibronectin isoform by preferential alternative splicing of a previously unobserved exon. *Embo J* 1987, 6(8):2337-2342.

**Tables**
